# NullSeq: A Tool for Generating Random Coding Sequences with Desired Amino Acid and GC Contents

**DOI:** 10.1371/journal.pcbi.1005184

**Published:** 2016-11-11

**Authors:** Sophia S. Liu, Adam J. Hockenberry, Andrea Lancichinetti, Michael C. Jewett, Luís A. N. Amaral

**Affiliations:** 1 Department of Chemical and Biological Engineering, Northwestern University, Evanston, Illinois, United States of America; 2 Interdisciplinary Program in Biological Sciences, Northwestern University, Evanston, Illinois, United States of America; 3 Northwestern Institute on Complex Systems, Northwestern University, Evanston, Illinois, United States of America; 4 Chemistry of Life Processes Institute, Northwestern University, Evanston, Illinois, United States of America; 5 Department of Physics and Astronomy, Northwestern University, Evanston, Illinois, United States of America; University of Chicago, UNITED STATES

## Abstract

The existence of over- and under-represented sequence motifs in genomes provides evidence of selective evolutionary pressures on biological mechanisms such as transcription, translation, ligand-substrate binding, and host immunity. In order to accurately identify motifs and other genome-scale patterns of interest, it is essential to be able to generate accurate null models that are appropriate for the sequences under study. While many tools have been developed to create random nucleotide sequences, protein coding sequences are subject to a unique set of constraints that complicates the process of generating appropriate null models. There are currently no tools available that allow users to create random coding sequences with specified amino acid composition and GC content for the purpose of hypothesis testing. Using the principle of maximum entropy, we developed a method that generates unbiased random sequences with pre-specified amino acid and GC content, which we have developed into a python package. Our method is the simplest way to obtain maximally unbiased random sequences that are subject to GC usage and primary amino acid sequence constraints. Furthermore, this approach can easily be expanded to create unbiased random sequences that incorporate more complicated constraints such as individual nucleotide usage or even di-nucleotide frequencies. The ability to generate correctly specified null models will allow researchers to accurately identify sequence motifs which will lead to a better understanding of biological processes as well as more effective engineering of biological systems.

## Author Summary

The generation of random sequences is instrumental to the accurate identification of non-random motifs within genomes, yet there are currently no tools available that allow users to simultaneously specify amino acid and GC composition to create random coding sequences. Here, we develop an algorithm based on maximum entropy that consistently generates fully random nucleotide sequences with the desired amino acid composition and GC content.

## Introduction

Genome sequencing costs continue to decline, resulting in a vast increase in the number of publicly available genome sequences spanning a wide range of diverse species. The exponential increase of sequencing data has lead to the development of computational pipelines, software tools, and algorithms to perform tasks such as genome annotation [1–4], phylogenetic inference [5, 6], and protein structure analysis [7–9] using only nucleotide sequences. All of these tools must be able to separate patterns within genome sequences from random noise.

The enrichment or depletion of certain nucleotide patterns in parts of the genome may provide vital information regarding different biological processes. For instance, the TATAAT and TTGACA motifs [10] and the Shine-Dalgarno sequence [11] are just some of the nucleotide patterns that occur upstream of prokaryotic coding sequences and bind to the RNA polymerase or ribosome to initiate transcription and translation. However, they are all depleted in the coding sequence [12], presumably to avoid initiation at improper sites. Motifs recognized by restriction enzymes are also significantly depleted within host genomes, decreasing the chance of accidental cleavage of the native DNA of organisms expressing these enzymes [12]. Additionally, it was recently shown that protospacer adjacent motifs (PAM) are depleted in phages that infect hosts with the CRISPR system because evolution selects against phages with motifs that are recognized by the bacteria’s innate defense [13].

The occurrence of specific motifs is also heavily influenced by global patterns such as GC content bias [14–17], di-nucleotide bias [18–20], codon bias [21–25], and codon pair bias [26, 27]. These higher-order patterns are the result of the combined effects of mutational biases and selection for accurate and efficient translation [23]. For example, species vary dramatically in the nucleotide composition of their genomes with individual bacterial species varying from 20-72% GC content [28, 29]. These patterns are thought to be the result of biased mutation rates [17] and/or selection for particular environments [30]. Regardless of the ultimate source of GC content variation, nucleotide composition is an important parameter to consider when assessing the over- or under-representation within the genome of motifs for a particular organism.

Analysis and identification of motifs relies heavily on the ability to computationally generate representative null models. Generating a random sequence of As, Ts, Gs, and Cs is trivial, and may be an effective null model to evaluate sequence motifs in intergenic regions of the genome. However, protein coding sequences are subject to more complex constraints, and an approach that ignores these constraints will result in erroneous results. A more realistic null model would not only need to eliminate all occurrences of stop codons, but also take into account other aspects of the gene such as its primary amino acid sequence and nucleotide usage frequencies. These important properties are intricately connected to the function of the protein in the host organism and should be considered when evaluating motif over- or under-representation in genomes.

Currently, there are several tools that enable users to generate random sequences with various constraints. Two of the most popular, SMS [31] and FaBox [32] allow users to create random coding sequences given a specific translation table and GC content, respectively. However, neither of these tools allow users to specify the amino acid usage of the translated sequence, making them inappropriate tools to evaluate specific genes or genomes. An alternative tool—GenRGenS—uses Markov Chains to create random coding sequences with similar poly-nucleotide usage for the evaluation of structural motifs [33]. However, these sequences, while having correct amino acid usage, fail to account for variations in GC content. To our knowledge, there are currently no tools available to create random sequences that simultaneously take into account amino acid usage and GC content constraints.

Here, we introduce a software tool, which we have named NullSeq, that allows users to generate random coding sequences with pre-specified amino acid usage and GC content. We show that previous heuristic methods that claim to be able to accomplish this goal fall short and actually produce sequences with GC contents that are almost always close to 50%. Our algorithm, which is based on a maximum entropy framework, can reliably produce random coding sequences based on the GC and amino acid content extracted from an existing sequence.

To describe the principle of maximum entropy, it is useful to briefly discuss its development in the 1950s in relation to statistical mechanics. Statistical physicists are frequently looking to connect macroscale properties of a system to microscale phenomenon. However, there may be a large, or even infinite, number of possible distributions for unobservable quantities (position and velocities of individual atoms) that are consistent with a few observable constraints on the system (temperature or density). In developing the principle of maximum entropy, Edward T. Jaynes noted that the existing mathematical formulation of this problem from statistical mechanics could be made into a more general statistical principle that has since found widespread applicability in numerous disciplines including biology [34, 35]. Given a set of constraints, the probability distribution with the largest information entropy will best represent the current state in the most unbiased manner [36, 37]. Specifically, given a set of observable nucleotide-level constraints, such as the GC content of the sequence, then the most *unbiased* distribution of nucleotides in the sequence is that which maximizes its informational entropy.

Using the principle of maximum entropy, our tool will aid researchers in creating more accurate null models for the purpose of finding sequence motifs that appear in genetic sequences. We have made NullSeq available online at https://github.com/amarallab/NullSeq.

## Results

### Assessing the Performance of Current Models

Creating random amino acid sequences with a specified amino acid composition can, in principle, be easily accomplished if we imagine a bag of marbles in which the number of marbles of a given color in the bag is proportional to the frequency of the corresponding amino acid in a desired sequence. Drawing at random *n* marbles out of the bag (with replacement) yields a “protein sequence” of length *n*. Creating a random nucleotide sequence from this primary amino acid sequence would then involve choosing one of the synonymous codons for each amino acid with uniform probability.

Unfortunately, this naïve approach provides no control over the GC content of the resulting nucleotide sequence. The GC content of sequences generated according to this method will be Gaussian distributed around a value that depends entirely on the amino acid composition of the sequence (S1 Fig).

One way to generate sequences that fulfill both amino acid and GC content constraints is to generate random sequences as above but only select the ones that are within the desired range of GC contents. However, in addition to being tremendously slow and computationally inefficient, this method yields sequences with a non-Gaussian GC content distribution. This is because sequences that have a GC content closer to the mean of the Gaussian distribution will occur more frequently, resulting in the large majority of the accepted sequences having a GC content that is biased towards the bounds of the acceptance range.

Controlling the nucleotide content of the sequence for a given protein in an efficient and unbiased manner requires manipulating the probability of choosing synonymous codons. A common implementation assumes that the probability of using synonymous codons is proportional to the target GC content, which we refer to as as the multinomial method [38, 39] (see Methods). That is, if the target sequence is to have twice as many Gs and Cs as As and Ts, then the probability of choosing the synonymous codon with the G/C nucleotide will be double that of the probability of choosing the synonymous codon with the A/T nucleotide.

**Fig 1:**
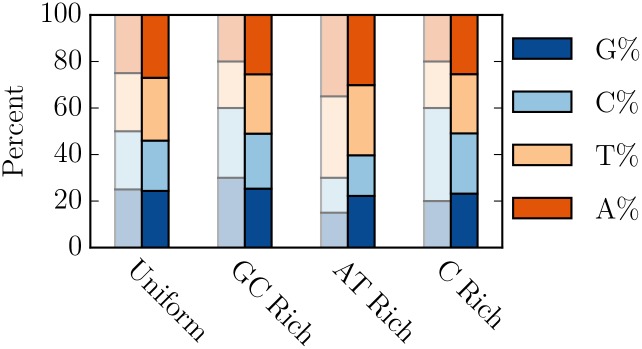
The multinomial method does not generate random sequence with the desired nucleotide composition. We tested the accuracy of the multinomial method by generating 500 sequences that were 2500 amino acid long, with uniform amino acid usage with four different target nucleotide contents (unsaturated color). Our results (saturated color) demonstrate that the multinomial method is unable to attain the specified individual nucleotide composition and also unable to attain the desired GC content.

In order to test whether the multinomial method is capable of creating genes with a target GC content, we generated 500 independent sequences, 2500 amino acids in length, for four different nucleotide compositions (Table 1). We found that the nucleotide composition of the resulting random sequences does not correspond to the desired nucleotide composition (Fig 1). Furthermore, the random sequences generated in this manner also fail at the simpler goal of specifying GC content.

**Table 1:** Target nucleotide composition of test sequences.

Nucleotide Usage	G%	C%	A%	T%
Uniform	25	25	25	25
GC Rich	30	30	20	20
AT Rich	15	15	35	35
C Rich	20	40	20	20

### Maximum Entropy Approach

While specifying the frequency of individual nucleotides is useful, we first sought to tackle the simpler problem of being able to specify the GC content of a given amino acid sequence. Fortunately, figuring out how much more likely one synonymous codon should occur over the others given the total number of G/C nucleotides in the sequence can be determined using the principle of maximum entropy. Following this approach, the probability of observing a particular synonymous codon given its amino acid is:

$$\begin{array}{c} P\left(C\right)=e^{-\beta E_{C}}/Z \end{array}$$ (1)

where *E*_*C*_ is the number of G/C nucleotides in codon *C*, *Z* typically denotes the partition function or a normalization constant such that the probabilities of synonymous codons sums to 1, and *β* is a variable related to the total number of G/C nucleotides in the sequence. Using this probability we can determine the expected number of G/C nucleotides for an amino acid sequence as a function of *β*,

$$\begin{array}{c} \frac{N}{Z}\sum_{a=1}^{20}(f\left(a\right)\sum_{C=1}^{m(a)}E_{C}e^{-\beta E_{C}})=E\left[n_{gc}\right] \end{array}$$ (2)

where *N* is the number of amino acids in the sequence, *f*(*a*) is the frequency of amino acid *a* in the sequence, *m*(*a*) is the degeneracy number of amino acid *a*, and *n*_*gc*_ is the number of G/C nucleotides in the sequence

Because the total number of G/C nucleotides of an amino acid sequence is strictly a function of the variable *β*, solving for the *β* that satisfies Eq (2) with the desired GC content will provide the synonymous codon usage probability that will yield sequences that fulfill the GC content constraints (see Methods for more details).

**Fig 2:**
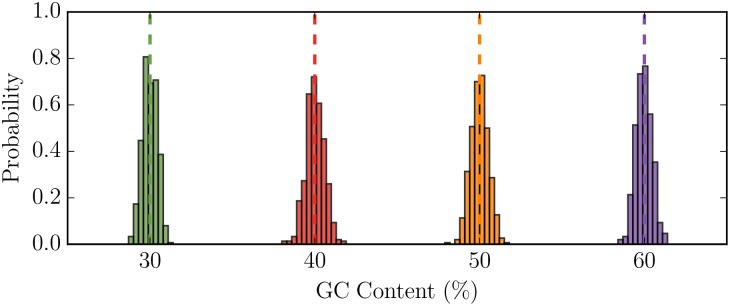
Random sequences generated using the maximum entropy approach are unbiased with a mean equal to the target GC content. We generated 500 random sequences, with equiprobable amino acid usage and 2500 amino acids in length. We used matching colors for target GC content (dashed line) and observed GC content distribution.

We again generated 500 random sequences each 2500 amino acids in length for four different target GC ratios: 0.3, 0.4, 0.5, and 0.6. Using the maximum entropy approach, we found that all the random sequences generated matched the desired GC content and are Gaussian distributed with a mean equal to the target GC content (Fig 2) indicating that the method is unbaised.

**Fig 3:**
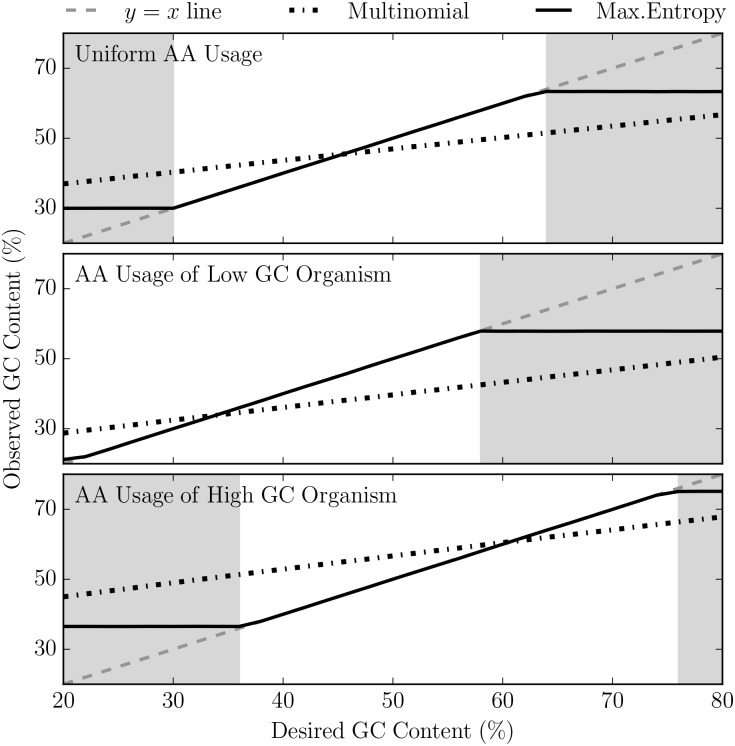
The GC ratio of random sequences generated using the maximum entropy approach coincides exactly with desired GC content over a wide range of GC ratios. When generating nucleotide sequences from an amino acid sequence with uniform amino acid usage, we can accurately achieve a GC content between the range of 30% and 64% (top). By altering the amino acid composition of the translated sequence, a lower and higher range of GC contents can be obtained (middle and bottom). At each GC content, the average GC content of 500 randomly generated sequences with amino acid length of 2500 was taken. The *y* = *x* line (shown in gray dotted line) indicates the ideal case. The simulated results for the multinomial and maximum entropy method are shown in black jagged and solid lines respectively.

To determine the limits of our method, we generated sequences across a range of GC contents from 20% to 80%. For sequences with uniform amino acid usage, our method reliably generates sequences in the range of 30% to 64% (Fig 3). Our method is unable to generate sequences with desired GC compositions beyond these limits due to the fact that the genetic code expressly prohibits certain amino acid usages and GC content combinations. This is actually consistent with the fact that organisms with extreme GC contents must have altered amino acid usage frequencies in their proteome [40, 41].

To show that our method can reliably generate random sequences with extreme GC contents, we used our maximum entropy approach to generate random sequences with the amino acids frequencies matching those of low and high GC organisms (*Anaeromyxobacter dehalogenans* and *Streptomyces coelicolor*, respectively) (S1 Table). In this way we were able to generate sequences with desired GC contents as low as 20% and as high as 76%.

## Discussion

The ability to generate unbiased random nucleotide sequences is very important for the investigation and discovery of biologically relevant motifs in a genome that may contribute to important biological processes. In order to correctly identify these nucleotide patterns, appropriate null models must be used. Especially for the evaluation of protein coding sequences, additional constraints must be imposed on the random sequences so that they are representative of the test sequence, such as stop codon depletion, nucleotide content, and amino acid usage. Previous heuristic methods used to generate random sequences that simultaneously constrain nucleotide and amino acid content have been shown to be biased and thus unable to generate sequences obeying the desired constraints.

Using the principle of maximum entropy, we successfully developed a method that is able to generate random nucleotide sequences with pre-specified amino acid frequencies or primary amino acid sequence subject to GC content constraints. Here we show that our method yields sequences that (1) are Gaussian distributed with the mean at the desired GC content (Fig 2) and (2) coincides with the desired GC content over a biologically relevant range (Fig 3).

While we have only explicitly derived the method to constrain GC content, the maximum entropy framework endows it with the ability to be easily mailable to additional or looser constraints. For example, the proposed method can be easily modified to allow for specification of a range of allowable GC contents. Under such conditions, the random sequences generated will exhibit a nearly uniform distribution along a portion of the allowable GC range followed by a fast decaying tail (S2 Fig). But even this can be addressed. In order to generate uniform distribution over the entire allowable GC range one can expand the sampling range on both sides and disregard sequences that do not fall in the desired GC content range (S3 Fig).

Additionally, more constraints can also be easily incorporated by adding to the number of variables that need to be simultaneous solved for in Eq (7). These additional constraints could be properties such as individual nucleotide content or di-nucleotide content which will allow researches to further expand on the types of nucleotide patterns that can be evaluated. However, it should be noted additional constraints will also increase the mathematical complexity of the problem, and significantly increase the computational time required to generate the sequences.

## Methods

### Multinomial Method Details

The most general form for calculating the probability of using one of its synonymous codon (*C*_*i*_) for an amino acid is:

$$\begin{array}{c} P\left(C_{i}\right)=\frac{1}{Z}\prod_{j=1}^{3}f\left(n_{j}\right) \end{array}$$ (3)

where *f*(*n*_*j*_) is the probability of using the nucleotide in the *j*th position and Z is a normalization factor so that the sum of the probabilities of synonymous codons is 1.

For example, if the goal is to create a nucleotide sequence that is 30% Gs, 30% Cs, 20% As, and 20% Ts, then for each phenylalanine in the amino acid sequence, TTT and TTC will be used with a probability of 0.4 and 0.6, respectively.

### Maximum Entropy Method Details

Given a set of *L* discrete states, {*S*_*k*_ : *k* = 1, …, *L*}, each with *M* observable properties, the set of all observable properties of the system is {*X*_*j*,*k*_ : *X*_*j*,*k*_ = *f*_*j*_(*S*_*k*_), *j* = 1, …, *M*, *k* = 1, …, *L*,} and the expected value of any observable for the entire system is:

$$\begin{array}{c} E\left[X_{j}\right]=\sum_{k=1}^{L}p\left(S_{k}\right)f_{j}\left(S_{k}\right)=\sum_{k=1}^{L}p\left(S_{k}\right)X_{j,k} \end{array}$$ (4)

where *p*(*S*_*k*_) is the probability of observing state *S*_*k*_.

Any observable property of the system can be constrained by specifying its total value in the system (*A*_*j*_), which implies:

$$\begin{array}{c} E\left[X_{j}\right]=A_{j} \end{array}$$ (5)

The {*p*(*S*_*k*_):*k* = 1, …, *L*} that simultaneously satisfies the set of equations in Eq (5) is the probability of observing each state given the specified constraints.

Using the principle of maximum entropy, this probability is:

$$\begin{array}{c} p\left(S_{k}\right)=\frac{1}{Z}\prod_{j=1}^{M}e^{\beta_{j}X_{j,k}} \end{array}$$ (6)

where *Z* is a normalization constant so that $\sum_{k=1}^{L}p(S_{k})=1$ and *β*_*j*_ is a tunable variable to ensure that Eq (5) is satisfied [34]. Eqs (4)–(6) can be combined to yield:

$$\begin{array}{c} \sum_{k=1}^{L}\frac{E_{j,k}}{Z}\prod_{j\prime =1}^{M}e^{\beta_{j\prime }X_{j\prime ,k}}=A_{j} \end{array}$$ (7)

Determining the correct probabilities {*p*(*S*_*k*_)} that satisfy the constraints {*A*_*j*_}, means solving for {*β*_*j*_} in Eq (7). As {*p*(*S*_*k*_)} is strictly a function of {*β*_*j*_}; determining {*β*_*j*_} will give you {*p*(*S*_*k*_)}, which is the probability of seeing each state that will yield the maximally unbiased composition of the states in the system given the imposed constraints.

In the context of creating random sequences with specified amino acid and GC constraints, each codon (C) will be a state and the observable property is the number of G/C nucleotides (*n*_*gc*_) in the sequence. Here, we want to determine the probabilities of using each codon given that the total number of G/C nucleotides is subject to constraint(*N*_*gc*_). Eq (4) now can be rewritten as Eqs (2) and (5) as:

$$\begin{array}{c} E\left[n_{gc}\right]=N_{gc} \end{array}$$ (8)

In this problem, we are only imposing a single constraint on the system (*N*_*gc*_). Unlike the general equations above where *j* variables need to be simultaneously solved for from the systems of equations in Eq (7), for this problem, only one variable needs to be determined, greatly simplifying the problem at hand. From Eq (2), $E[n_{gc}]$ (and thus GC content) is a function of only *β* because the amino acid frequencies and the length of the sequences are all known values (S3 Fig). *β* is solved for numerically, so that Eq (8) is satisfied.

After determining the value of *β*, {*P*(*C*_1_, …, *P*(*C*_61_)} can be calculated using Eq (1). The random nucleotide sequence is then generated from a primary amino acid sequence (either previously defined or generated randomly according to specified amino acid frequencies) by choosing synonymous codons with the normalized probabilities defined by {*P*(*C*_1_, …, *P*(*C*_61_)} for each amino acid in the sequence.

## Supporting Information

S1 Fig Expected GC content of random sequences depends on amino acid usage if synonymous codons are chosen with uniform probability. The histogram shows the GC content distribution for three different amino acid usage frequencies, from a high GC organism (*Streptomyces coelicolor*), a low GC organism (*Anaeromyxobacter dehalogenans*), and uniform usage. The mean GC ratios of the random sequences are 0.57, 0.40, and 0.47, respectively.
(PDF)

S2 Fig Distribution of the GC contents of random sequences when an allowable GC content range is specified. When a the desired GC content is set to a range instead of a singular value, the GC content distribution for the random sequences will be uniform within most of GC content range with a decaying tail at both ends (top). To get a uniform distribution within an entire desired range, the range can be expanded slightly so that the desired GC range is encompassed within the portion that exhibits a uniform distribution and any sequences that do not fall within the GC range is thrown out (bottom). For each example, we generated 50000 random sequences, with equiprobable amino acid usage and 2500 amino acids in length. The dashed blue lines correspond to the minimum (40%) and maximum (45%) allowable GC content.
(PDF)

S3 Fig The dependence of GC content on *β* given amino acid usage frequencies. For a given amino acid usage frequency, the GC content of the generated sequence will depending on the values of *β*. Low values of *β* will yield sequences will higher GC content, and vice versa. The GC content of the sequence is also dependent on the amino acid usage frequency of the sequence due to the number of G/C nucleotides in its codons. With the same *β*, the resulting GC content of the sequence will change depending on the amino acid usage frequency.
(PDF)

S1 Table Amino acid usage probabilities. The high GC content organism is *Streptomyces coelicolor* and the low GC content organism is *Anaeromyxobacter dehalogenans*.
(PDF)
