# Supplementary figures and images for: NullSeq: A Tool for Generating Random Coding Sequences with Desired Amino Acid and GC Contents
Source: PLoS Comput Biol. 2016 Nov 11;12(11):e1005184. doi: 10.1371/journal.pcbi.1005184 (PMC5106001; doi:10.1371/journal.pcbi.1005184)

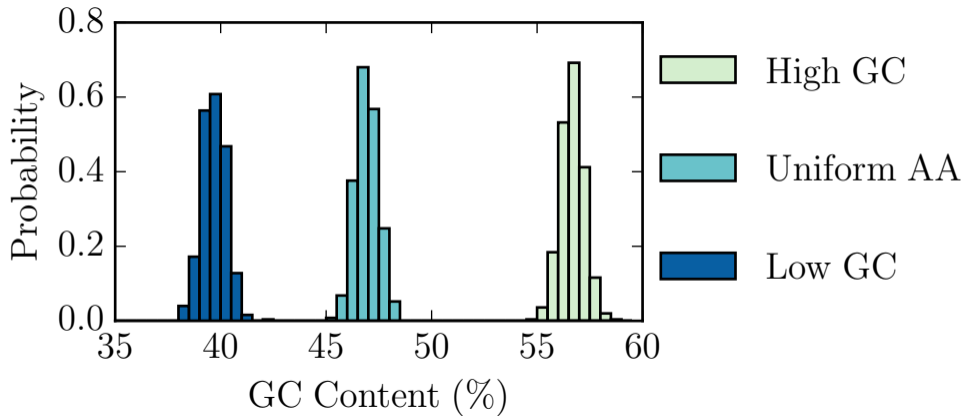

Supplement: S1 Fig — The histogram shows the GC content distribution for three different amino acid usage frequencies, from a high GC organism (Streptomyces coelicolor), a low GC organism (Anaeromyxobacter dehalogenans), and uniform usage. The mean GC ratios of the random sequences are 0.57, 0.40, and 0.47, respectively. (PDF) [file pcbi.1005184.s001.pdf]

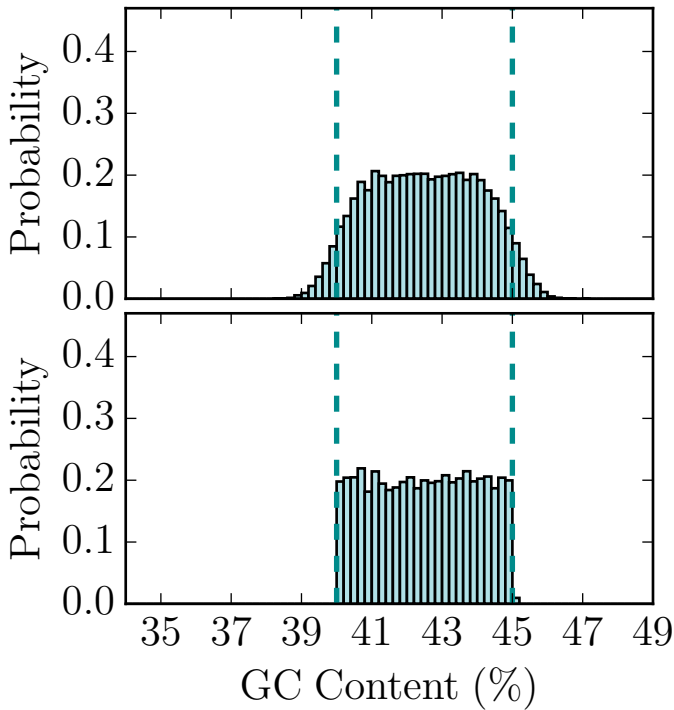

Supplement: S2 Fig — When a the desired GC content is set to a range instead of a singular value, the GC content distribution for the random sequences will be uniform within most of GC content range with a decaying tail at both ends (top). To get a uniform distribution within an entire desired range, the range can be expanded slightly so that the desired GC range is encompassed within the portion that exhibits a uniform distribution and any sequences that do not fall within the GC range is thrown out (bottom). For each example, we generated 50000 random sequences, with equiprobable amino acid usage and 2500 amino acids in length. The dashed blue lines correspond to the minimum (40%) and maximum (45%) allowable GC content. (PDF) [file pcbi.1005184.s002.pdf]

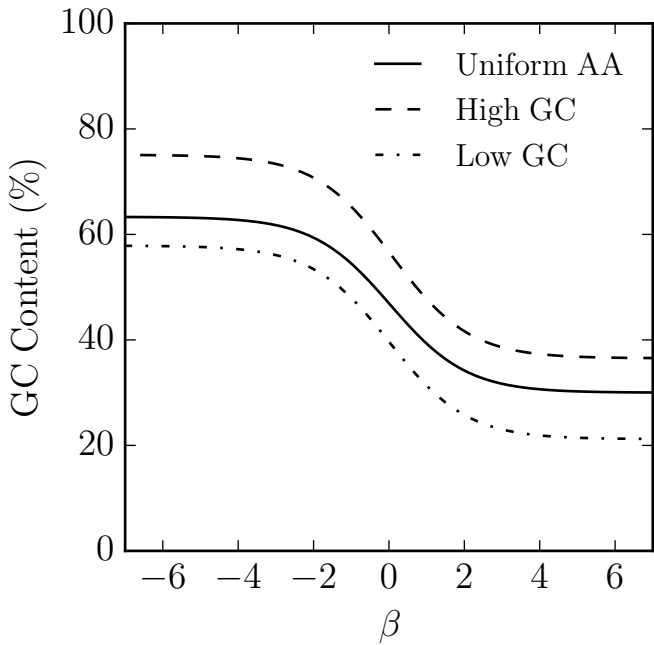

Supplement: S3 Fig — For a given amino acid usage frequency, the GC content of the generated sequence will depending on the values of β. Low values of β will yield sequences will higher GC content, and vice versa. The GC content of the sequence is also dependent on the amino acid usage frequency of the sequence due to the number of G/C nucleotides in its codons. With the same β, the resulting GC content of the sequence will change depending on the amino acid usage frequency. (PDF) [file pcbi.1005184.s003.pdf]
