# Supplementary material for: NullSeq: A Tool for Generating Random Coding Sequences with Desired Amino Acid and GC Contents
Source: PLoS Comput Biol. 2016 Nov 11;12(11):e1005184. doi: 10.1371/journal.pcbi.1005184 (PMC5106001; doi:10.1371/journal.pcbi.1005184)
Supplement: S1 Table — The high GC content organism is Streptomyces coelicolor and the low GC content organism is Anaeromyxobacter dehalogenans. (PDF) [file pcbi.1005184.s004.pdf]

S1 Table. Amino acid usage probabilities

AA	Probability (%)	
	Streptomyces coelicolor	*Anaeromyxobacter dehalogenans*
N	0.0170	0.0846
E	0.0571	0.0609
W	0.0153	0.0104
R	0.0840	0.0252
V	0.0855	0.0540
S	0.0500	0.0680
L	0.1025	0.1001
D	0.0616	0.0569
Q	0.0267	0.0378
T	0.0620	0.0534
H	0.0237	0.0131
I	0.0288	0.1006
M	0.0140	0.0144
K	0.0206	0.1083
P	0.0622	0.0246
C	0.0078	0.0059
A	0.1378	0.0451
F	0.0265	0.0526
G	0.0963	0.0431
Y	0.0206	0.0410
